# Fluorinated vs. Zwitterionic-Polymer Grafted Surfaces for Adhesion Prevention of the Fungal Pathogen *Candida albicans*

**DOI:** 10.3390/polym12020398

**Published:** 2020-02-10

**Authors:** Elena Masotti, Noemi Poma, Elisa Guazzelli, Ilenia Fiaschi, Antonella Glisenti, Federico Vivaldi, Andrea Bonini, Fabio Di Francesco, Arianna Tavanti, Giancarlo Galli, Elisa Martinelli

**Affiliations:** 1 Dipartimento di Chimica e Chimica Industriale and UdR Pisa INSTM, Università di Pisa, 56124 Pisa, Italy; elenam91@gmail.com (E.M.); vio_noemia15@hotmail.com (N.P.); elisa.guazzelli@for.unipi.it (E.G.); ileniafiaschi@outlook.it (I.F.); federico-vivaldi@virgilio.it (F.V.); andrea.bonini@phd.unipi.it (A.B.); fabio.difrancesco@unipi.it (F.D.F.); giancarlo.galli@unipi.it (G.G.); 2 Dipartimento di Scienze Chimiche, Università di Padova, 35131 Padova, Italy; antonella.glisenti@unipd.it; 3 Dipartimento di Biologia, Università di Pisa, 56126 Pisa, Italy; arianna.tavanti@unipi.it

**Keywords:** *Candida albicans*, zwitterionic polymer, fluorinated polymer, biofouling, antifouling, adhesion

## Abstract

Fluorinated (F6) and zwitterionic, as well as phosphorylcholine (MPC) and sulfobetaine (MSA), copolymers containing a low amount (1 and 5 mol%) of 3-(trimethoxysilyl)propyl methacrylate (PTMSi) were prepared and covalently grafted to glass slides by using the trimethoxysilyl groups as anchorage points. Glass-surface functionalization and polymer-film stability upon immersion in water were proven by contact angle and angle-resolved X-ray photoelectron spectroscopy (AR-XPS) measurements. Antifouling performance of the grafted films was assayed against the yeast *Candida albicans*, the most common *Candida* species, which causes over 80% of candidiasis. Results revealed that the F6 fluorinated, hydrophobic copolymers performed much better in reducing the adhesion of *C. albicans*, with respect to both corresponding zwitterionic, hydrophilic MPC and MSA counterparts, and were similar to the glass negative control, which is well-known to inhibit the adhesion of *C. albicans*. A composition-dependent activity was also found, with the films of copolymer with 99 mol% F6 fluorinated co-units performing best.

## 1. Introduction

Biofouling is generally an undesirable phenomenon that involves the organic matter deposition and/or organism colonization of surfaces upon contact with biological fluids, freshwater or seawater [1,2]. Thus, biofouling affects medical, industrial and marine fields, resulting in detriments to health and environment and increased operational costs [3,4,5]. In the medical field, biofilm-associated microorganisms are responsible for up to 80% of all microbial infections in humans [6,7]. Biofilms are linked to recurrent invasive infections that are difficult to eradicate because of their intrinsic resistance to antimicrobial treatments and host defense mechanisms and their excellent ability to adhere to biomaterials [8].

The increasing use of biomaterials and medical devices, such as catheters, stents, prostheses, contact lenses and implants, has led to a concomitant increase in the incidence of device-related infections, with the most common fungal infection due to *Candida albicans* [9,10]. Management of biofilm-associated *Candida* infections can be challenging due to the intrinsic drug-resistant phenotype of sessile fungal cells, and removal of the infected device is often required [9]. However, removal of the contaminated implant can be accompanied by complications, negatively affecting the patient’s condition and the economic burden. Therefore, prevention of biofilm-associated infections currently represents a major challenge.

The surface properties, notably the surface chemical composition, of materials susceptible to fouling are known to significantly affect the biofilm formation on medical devices [11], as well as the micro- and macro-fouler colonization of ship hulls, maritime equipment and industrial implants [12,13,14,15]. One of the most promising strategies to overcome this problem is to develop anti(bio)fouling surfaces, which prevent microorganism adhesion.

The first interactions occurring among *C. albicans* cells and materials surfaces are usually hydrophobic interactions and electrostatic forces, taking place within the first 12 h; subsequent stages involve a stronger adhesion, displayed by cell-wall glycoproteins (e.g., Als or Hwp1 proteins) [16]. This leads to formation of microcolonies (3–4 h) and biofilm aggregates organized in a bilayer composed of yeast and hyphal cells embedded in a self-produced extracellular polymeric matrix (11–30 h). *C. albicans* mature biofilm is then consolidated up to 38–72 h [17].

Among the different parameters that are reported to possibly affect *Candida* adhesion, including surface preconditioning with different biological fluids [12], surface roughness [8,9,10,11] and surface charge [9], one of the most relevant is surface wettability. It is widely acknowledged that *C. albicans* attaches more rapidly to hydrophobic, nonpolar surfaces, such as Teflon and other plastics, than to hydrophilic surfaces, such as glass and metals [18]. However, results of these studies have at times been contradictory, due to the absence of standardized methods for determining surface hydrophobicity, and the wide variability observed between different *Candida* species, as they may show differences in the cell-wall composition [19]. A considerable extent of variability in adhesive properties has also been described even among *C. albicans* isolates [20]. For instance, Kang et al. evaluated the effect of surface properties of four different types of denture-lining material (tissue conditioners, acrylic and silicone soft liners and hard reline materials) on the adhesion of *C. albicans*. Surface-energy parameters of the different materials were evaluated, and the data obtained indicated that acrylic soft liners were more hydrophilic than other materials and, together with tissue conditioners, displayed greater *Candida* adhesion than did silicone soft liners and hard reline materials [21].

Since hydrophobic interactions appear to play a key role in the adhesion of *C. albicans* to prosthetic materials [10], hydrophilic surface modification of biological devices has been regarded as an effective strategy to inhibit *C. albicans* colonization. Attempts have been performed to modify denture-base materials to make them more hydrophilic. In particular, Lazarin et al. coated denture-base acrylic resins with photopolymerized coatings containing zwitterionic or hydrophilic monomers and found out that those based on sulfobetaine methacrylate and 3-hydroxypropyl methacrylate significantly reduced the adhesion of *C. albicans* with respect to the untreated acrylic [22,23]. Superhydrophilic sulfobetaine-methacrylamide-based copolymers on denture surfaces were shown to enhance the hydrophilicity of the denture-base acrylic resin and reduce the initial adhesion of *C. albicans* [24]. Inorganic silica was also used for hydrophilic modification of acrylic denture resins, to reduce the colonization of *C. albicans* [25]. Acrylic resin surfaces were also modified by different plasma treatments, including argon, argon/oxygen and argon/sulfur hexafluoride atmospheres, in order to obtain surfaces with different wettability. Interestingly, it was found that both the hydrophobic Ar/SF_6_ and the hydrophilic Ar/O_2_ treated resins were able to significantly reduce the adhesion of *C. albicans*, regardless of the different initial contact angle values, the presence or absence of saliva and the surface roughness [26]. Silicone rubber is also used for oral cavity applications, including voice prostheses and denture soft-liners, and its surface modification with fluorinated trialkoxysilane has been proposed to enhance the resistance of silicone medical devices to *C. albicans* adhesion [27].

The present work aimed to better understand the effect of surface phobicity/philicity against the adhesion of the yeast pathogen *C. albicans* on hydrophobic, fluorinated polymers, as opposed to hydrophilic, zwitterionic polymers. In particular, we synthesized three sets of copolymers based on a hydrophobic fluorinated (F6) monomer, a hydrophilic zwitterionic, phosphorylcholine (MPC) or sulfobetaine (MSA), monomer with 3-(trimethoxysilyl)propyl methacrylate (PTMSi). The latter was incorporated to a minimal amount (1 and 5 mol%), to ensure covalent anchoring of the polymer film to a glass substrate. The copolymers were then used to produce films whose surfaces were investigated by contact angle and angle-resolved X-ray photoelectron spectroscopy measurements, both before and after immersion in water, to characterize the chemical structure of the polymer surface. Adhesion of *C. albicans* to the chemically different polymer films was assessed and related to the surface phobic/philic chemical nature. To the best of our knowledge, this is the first paper where the antifouling properties of zwitterionic and fluorinated copolymers against the yeast pathogen *C. albicans* are directly compared.

## 2. Materials and Methods

### 2.1. Materials

1*H*,1*H*,2*H*,2*H*-Perfluorooctyl acrylate (F6, Fluorochem, Hadfield, UK), 3-(trimethoxysilyl)propyl methacrylate (PTMSi, ABCR, Karlsruhe, Germany), *N*-(3-sulfopropyl)-*N*-(methacryloyloxyethyl)-*N*,*N*-dimethylammonium betaine (MSA, Sigma Aldrich, St. Louis MO, USA), 2-methacryloyloxyethyl phosphorylcholine (MPC, Sigma Aldrich, St. Louis MO, USA), 2,2′-azobis(2-methylpropionamidine) dihydrochloride (V50, Sigma Aldrich, St. Louis MO, USA), hexafluorobenzene (HFB, Fluorochem, Hadfield, UK), *α*,*α*,*α*-trifluorotoluene (TFT, Fluorochem, Hadfield, UK) and 2,2,2-trifluoroethanol (TFE, Sigma Aldrich, St. Louis MO, USA) were used as received. 2,2′-Azobis(isobutyronitrile) (AIBN, Sigma Aldrich, St. Louis MO, USA) was recrystallized from methanol. Toluene was kept over CaH_2_ for 4 h and then distilled. Methanol was kept over molecular sieves before use. Tetrahydrofuran (THF) was refluxed and distilled on Na/K.

### 2.2. General Procedure for the Synthesis of Zwitterionic Copolymers p(MSA-co-PTMSix)

In a typical preparation, monomers MSA (0.991 g, 3.54 mmol) and PTMSi (10 µL, 0.04 mmol), initiator V50 (10 mg) and anhydrous methanol (4 mL) were introduced into a Carius tube. The solution was outgassed by four freeze–pump–thaw cycles. The polymerization reaction was allowed to proceed under stirring at 50 °C for 6 h. The crude product was purified by several precipitations from TFE into methanol (yield 33%). The obtained copolymer p(MSA-*co*-PTMSi1) contained 99 mol% MSA and 1 mol% PTMSi.

^1^H NMR (in D_2_O/TFE, δ ppm): 4.60–4.40 (COOCH_2_), 3.76–3.56 (CH_2_N^+^CH_2_, OCH_3_), 3.45–3.16 (CH_2_N^+^CH_2_, N^+^(CH_3_)_2_), 3.14–2.93 (CH_2_SO_3_^–^), 2.45–2.21 (CH_2_CH_2_SO_3_^–^), 2.18–1.90 (CH_2_), 1.40–0.70 (CH_3_, CH_2_Si).

### 2.3. General Procedure for the Synthesis of Zwitterionic Copolymers p(MPC-co-PTMSix)

In a typical preparation, monomers MPC (1.020 g, 3.46 mmol) and PTMSi (8 µL, 0.03 mmol), initiator V50 (10 mg) and anhydrous methanol (4 mL) were introduced into a Carius tube. The solution was outgassed by four freeze–pump–thaw cycles. The polymerization reaction was let to proceed under stirring at 50 °C for 24 h. The crude product was purified by several precipitations from methanol into THF (yield 85%). The obtained copolymer p(MPC-*co*-PTMSi1) contained 99 mol% MPC and 1 mol% PTMSi.

^1^H NMR (in D_2_O, δ ppm): 4.33 (COOCH_2_, MPC), 4.24 (CH_2_O(PO_2_^–^)OCH_2_)_,_ 4.12–3.90 (CH_2_O(PO_2_^–^)OCH_2_, COOCH_2_ PTMSi), 3.86–3.59 (CH_2_N^+^(CH_3_)_3_, OCH_3_), 3.27 (N^+^(CH_3_)_3_), 2.17–1.74 (CH_2_), 1.19–0.66 (CH_3_, CH_2_Si).

### 2.4. General Procedure for the Synthesis of Fluorinated Copolymers p(F6-co-PTMSix)

In a typical preparation, monomers F6 (1.27 mL g, 4.74 mmol) and PTMSi (19 µL, 0.08 mmol), initiator AIBN (20 mg) and anhydrous toluene (4 mL) were introduced into a Carius tube. The solution was outgassed by four freeze–pump–thaw cycles. The polymerization reaction was let to proceed under stirring at 70 °C for 24 h. The crude product was purified by several precipitations, from CHCl_3_/HFB into *n*-hexane (yield 60%). The obtained copolymer p(F6-*co*-PTMSi1) contained 99 mol% F6 and 1 mol% PTMSi.

^1^H NMR (in CDCl_3_/HFB, δ ppm): 4.47 (COOCH_2_ F6), 4.16–3.96 (COOCH_2_ PTMSi), 3.63 (OCH_3_), 2.77–2.37 (CH_2_CF_2_), 2.10 (CH), 1.83 (CH_2_ PTMSi, CH_2_CH_2_Si), 1.49 (CH_2_ F_6_), 1.25–1.00 (CH_3_), 0.73 (CH_2_Si).

^19^F-NMR (CDCl_3_/CF_3_COOH, δ ppm): –5 (CF_3_), –38 (CH_2_CF_2_), from –46 to –49 (CF_2_), –51 (CF_2_CF_3_).

### 2.5. Preparation of Polymer Films

Glass microscope slides (18 mm × 18 mm) were cleaned in hot piranha solution (concentrated sulfuric acid/30 wt% hydrogen peroxide, 7/3 *v*/*v*) for 1 h at 80 °C, rinsed with distilled water and kept under distilled water until use. Film deposition was carried out either by dip-coating or spin-coating.

#### 2.5.1. Dip-Coating

The clean glass slides were immersed for 2 h in a 5 wt% polymer solution of p(MSA-*co*-PTMSix) in TFE, p(MPC-*co*-PTMSix) in methanol and p(F6-*co*-PTMSix) in HFB, respectively. The coated slides were heated in an oven under vacuum, at 130 °C for 14 h, and then rinsed and sonicated for 15 min, in water (p(MSA-*co*-PTMSix) and p(MPC-*co*-PTMSix)) or TFT (p(F6-*co*-PTMSix)). The coated slides were finally heated at 130 °C for 1 h.

#### 2.5.2. Spin-Coating

Films for contact angle and XPS measurements were prepared by spin-coating. A 3 wt% polymer solution of p(MSA-*co*-PTMSix) in TFE, p(MPC-*co*-PTMSix) in methanol and p(F6-*co*-PTMSix) in HFB was spin-coated (8500 rpm, 20 s) on one side of the activated glass slides. Slides were then heated in an oven, under vacuum, at 130 °C, overnight, and then rinsed in water (p(MSA-*co*-PTMSix) and p(MPC-*co*-PTMSix)) or TFT (p(F6-*co*-PTMSix)). The coated slides were finally heated at 130 °C for 1 h.

### 2.6. Characterization

^1^H NMR and ^19^F NMR spectra were recorded with a Varian Gemini VRX300 spectrometer on CDCl_3_ and CDCl_3_/CF_3_COOH solutions, respectively.

Contact angles were measured by the sessile drop (10 µL) method with a FTA200 Camtel goniometer, using water (*θ*_w_) (J. T. Baker, HPLC grade) as wetting liquid. Average *θ*_w_ values of five measurements were reported.

X-ray photoelectron spectroscopy (XPS) spectra were recorded with a PerkinElmer PHI 5600 spectrometer with a standard Al–Kα source (1486.6 eV) operating at 350 W. The working pressure was less than 10^–8^ Pa. The spectrometer was calibrated by assuming the binding energy (BE) of the Au 4f_7/2_ line to be 84.0 eV, with respect to the Fermi level. Extended (survey) spectra were collected in the range 0–1350 eV (187.85 eV pass energy, 0.4 eV step, 0.05 s step^–1^). Detailed spectra were recorded for the following regions: C(1s), O(1s), Si(2p) and F(1s). The standard deviation (SD) in the BE values of the XPS line was 0.10 eV. The spectra were recorded at two photoemission angles *ϕ* (between the surface normal and the path taken by the photoelectrons) of 70° and 20°, corresponding to sampling depths of ~3 nm and ~9 nm (C(1s) line), respectively. The atomic percentage, after a Shirley-type background subtraction [28], was evaluated by using the PHI sensitivity factors (±1% experimental error) [29]. To take into account charging problems, the C(1s) peak was considered at 284.5 eV, and the peak BE differences were evaluated. The XPS peak-fitting procedure was carried out by means of Voigt functions, and the results were evaluated by an *χ*^2^ function [30].

Measurements of ζ potential were performed with a SurPass Anton Paar electrokinetic analyzer comprising a clamping cell for planar surfaces (26 cm × 76 cm) parallel to each other. The measurements were conducted by forcing a 0.001 M KCl electrolyte solution through the channel via syringe pumps. The pH of the electrolyte was adjusted from 10.0 to 2.5 stepwise with auto pH titration by adding 0.05 M HCl and 0.05 M NaOH. ζ potential was determined from streaming current data, using the Helmholtz–Smoluchowski equation [31]. An average of four measurements was used to represent each ζ potential value at any given pH.

### 2.7. Candida albicans Biofilm Formation Assay

#### 2.7.1. Strain

*Candida albicans* SC5314 was kindly provided by Professor Frank Odds, University of Aberdeen, UK. The reference strain SC5314 was stored in yeast peptone dextrose (YPD) broth (Difco BD, Milan, Italy) supplemented with 40% glycerol at −20 °C and −80 °C, and sub-cultured on YPD agar plates at 30 °C, when necessary. *C. albicans* inoculum was prepared from a single colony in YPD broth and incubated at 30 °C, overnight, under agitation.

#### 2.7.2. Biofilm Formation Assay

Coated glass slides were sterilized by exposure to UV light, 15 min by side. The concentration of each *C. albicans* suspension was microscopically determined, using a Bürker’s chamber and adjusted 2 × 10^7^ cells mL^–1^ in RPMI 1640 medium (supplemented with 2% glucose and morpholinepropanesulfonic acid (MOPS), pH 7) (Sigma Aldrich, St. Louis MO, USA). Slides were immersed in a 50 mL glass tube containing 7 mL of yeast suspension already prepared at the desired concentration in RPMI medium.

Cells were allowed to adhere to the polymer surface for 90 min at 37 °C, under agitation. Following incubation, non-adhered cells were removed by washing twice in phosphate-buffered saline (PBS) 1×. Then, 7.5 mL of RPMI 1640 medium (supplemented with 2% glucose and MOPS, pH 7) was added, and the glass cover slides were incubated at 37 °C for 24 h, in static conditions, to allow biofilm formation. Planktonic cells were removed by washing twice with PBS 1×. The slides were then transferred into a sterile 6 flat-bottomed-well microtiter plate, and 2 mL of PBS 1× was added to each well; biofilm-embedded cells were detached from the slide surface with a cell scraper. A replica set of each slide was used for microscopic analysis. The morphology of sessile cells was visualized under an inverted microscope (Olympus IMT-2) at 400× magnification.

Cells were transferred in a 2 mL tube, and cell aggregates were dissolved by sonication for 5 min and vortex for other 5 min. Cell dilutions were then prepared in PBS 1×, and then 200 µL was plated on a YPD agar plate and incubated at 30 °C overnight. Finally, individual colonies were counted, and results were expressed as colony-forming units (CFU) per slide.

All aforementioned polymer films were evaluated as substrates for fungal biofilm formation, a glass slide of the same area was included as a negative control. Three independent experiments were performed, each in triplicate. Results were expressed as means ± standard deviation. Statistical analysis was performed by using the Kruskal–Wallis test, followed by Dunn’s test, with a significance level of *p* ≤ 0.05.

## 3. Results and Discussion

### 3.1. Synthesis of Copolymers

**Figure 1:**
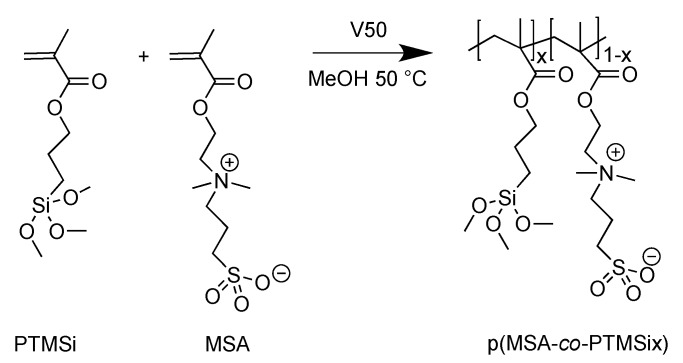
Synthesis of the zwitterionic copolymers based on hydrophilic MSA and PTMSi (x = 1, 5 mol%).

**Figure 2:**
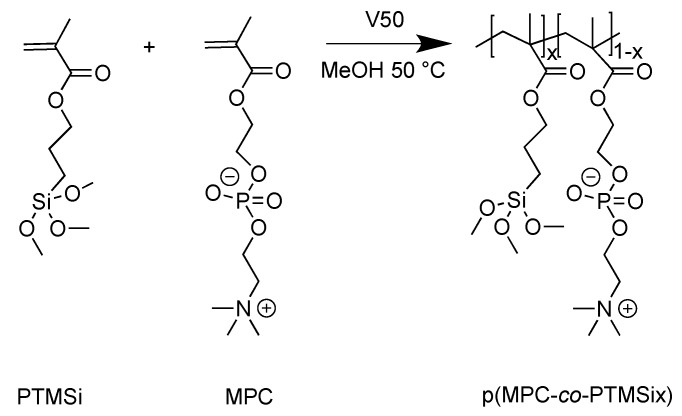
Synthesis of the zwitterionic copolymers based on hydrophilic MPC and PTMSi (x = 1, 5 mol%).

**Figure 3:**
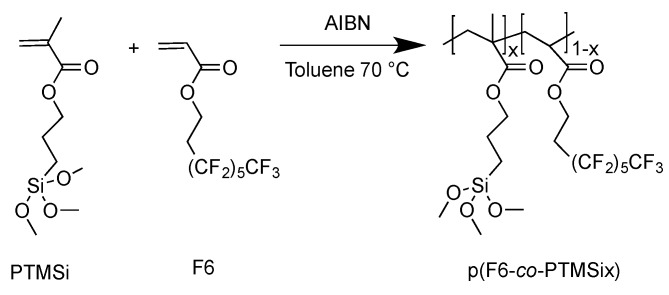
Synthesis of the fluorinated copolymers based on hydrophobic F6 and PTMSi (x = 1, 5 mol%).

Zwitterionic copolymers p(MSA-*co*-PTMSix) and p(MPC-*co*-PTMSix) based on hydrophilic MSA and MPC were synthesized by free-radical polymerization at 50 °C, by using anhydrous methanol as solvent (10 mL g^–1^ monomers) and V50 (1 wt%) as initiator (Figure 1 and Figure 2). V50 was chosen in place of more common thermal initiators, such as AIBN, owing to its higher solubility in methanol and its lower activation temperature (*t*_1/2_ = 13 h at 50 °C [32]). Fluorinated copolymers p(F6-*co*-PTMSix) based on hydrophobic (and lipophobic) F6 counterpart were synthesized in anhydrous toluene solution at 70 °C, by using AIBN (1 wt%) as free-radical initiator (Figure 3).

For any set of copolymers, an intentionally low amount of PTMSi counits (x = 1 and 5 mol%) was incorporated in the copolymer to warrant covalent anchorage of the copolymer to the glass surfaces through a “grafting-to” reaction during the polymer film preparation. The MPC-based copolymers were soluble in methanol, ethanol and water and poorly soluble in less polar solvents, such as THF and acetone. Therefore, they were purified by repeated precipitations from methanol solutions into THF. Differently, MSA-based copolymers were insoluble in ethanol, methanol and water and thus were purified by repeated precipitations from the very polar TFE) into methanol. The poor solubility of the MSA-based copolymers in water at room temperature was consistent with the known UCST behavior of the corresponding p(MSA) homopolymer. The fluorinated copolymers were purified by repeated precipitations from HFB solutions into hexane.

**Figure 4:**
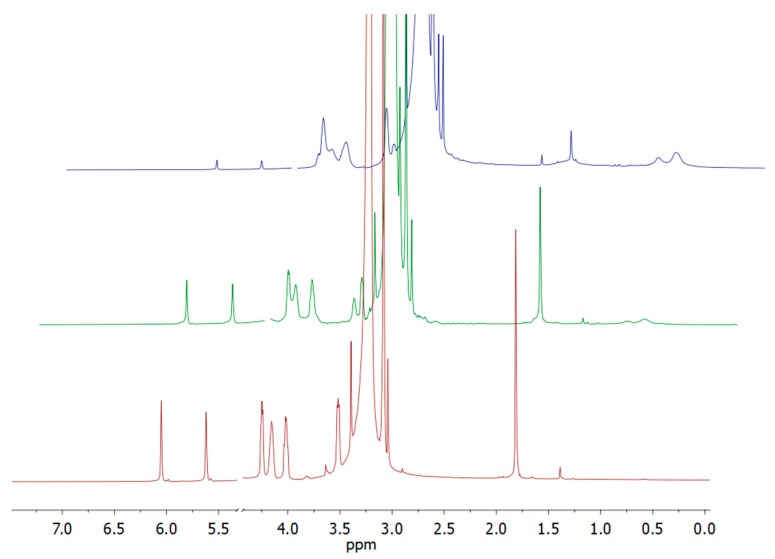
^1^H NMR spectra recorded at different reaction times (*t* = 0, 4 and 24 h from bottom to top) during the synthesis of copolymer p(MPC-*co*-PTMSi1).

The actual formation of copolymers was confirmed by ^1^H NMR and ^19^F NMR (p(F6-*co*-PTMSix)). The composition was calculated from the integral intensities of the signals of PTMSi at 3.6 ppm (OCH_3_) or 0.7 ppm (SiCH_2_) and the typical signals of the corresponding comonomers (4.47 ppm (COOCH_2_, F6), 3.27 ppm (N^+^(CH_3_)_3_), MPC) and 2.35 (CH_2_CH_2_SO_3_^–^, MSA)). The copolymerization runs were carried out up to almost complete conversion of monomers (95%–99%), which made the subsequent work-up steps much easier. The monomer conversion *p* was followed by ^1^H NMR spectroscopy (see Figure 4 for one illustration example) and calculated according to Equation (1):

*p* = 1 − *I*_(6.05 t=x)_/*I*_(6.05 t=0)_ (1)

where *I*_(6.05 t=*x*)_ and *I*_(6..05 t=0)_ are the integrals of the signals of vinyl proton at 6.05 ppm at different reaction times and at the initial time, respectively. At such high final comonomer conversions, the copolymer composition was equal to that of the respective monomer feed.

At the end of the polymerization, a suitable solvent (TFE, methanol and TFT for p(MSA-*co*-PTMSix), p(MPC-*co*-PTMSix) and p(F6-*co*-PTMSix), respectively) was added to dilute the copolymer solutions that were then stored under nitrogen at −20 °C. The molar masses and molar mass distributions of the copolymers could not be determined, owing to their poor solubility in the common solvents normally used for GPC analyses. Moreover, it was observed that the copolymers were difficult to handle because of the hydrolysis and sol-gel condensation of the Si(OCH_3_)_3_ groups of PTMSi counits.

### 3.2. Preparation of Polymer Films

**Figure 5:**
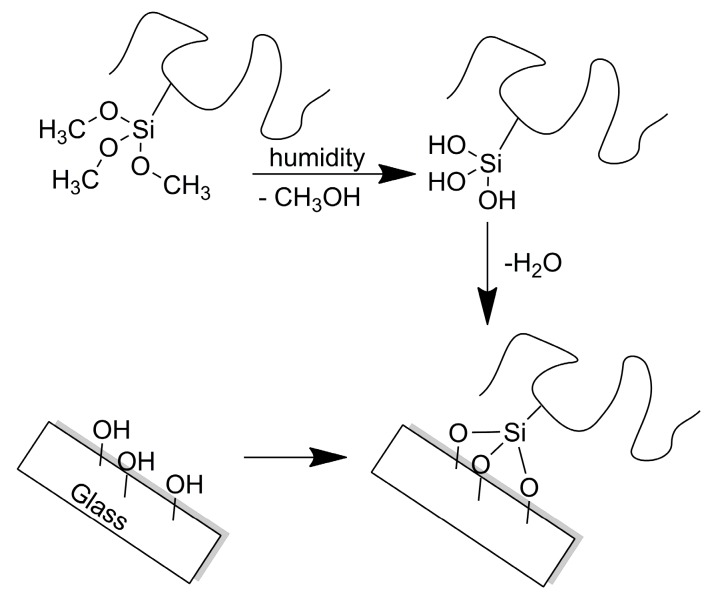
Schematic of the chemical functionalization of the glass slides with the zwitterionic and fluorinated copolymers containing PTMSi counits through a “grafting-to” approach.

Polymer films of the three different classes of copolymers were prepared via a “grafting-to” approach to covalently anchor the films to the glass surface, in order to prevent their delamination during biological assays, especially of those containing hydrophilic zwitterionic MSA and MPC. Before functionalization, glass slides were activated by immersion in hot piranha solution, thus cleaning the glass surface and forming additional silanol (Si–OH) groups at the outermost surface layers (~20 Å). The formation of such Si–OH groups was also inferred by the decreased water contact angle from 28 ± 1° to 20 ± 3° in going from the non-activated to the activated glass. After cleaning, the glass slides were stored in deionized water. The Si–OH groups were then exploited as anchorage points to bind the polymer films through a condensation sol-gel reaction with the silanol functionalities derived from the hydrolysis of the pendant PTMSi trimethoxysilyl groups promoted by ambient humidity (Figure 5). A 5 wt% copolymer solution was deposited by dip-coating on activated glass slides and thermally annealed at 130 °C in an oven, under vacuum, in order to remove the solvent and drive the cure reaction to completeness. The solvent of choice varied on the basis of the copolymer, being TFE, methanol and HFB for p(MSA-*co*-PTMSix), p(MPC-*co*-PTMSix) and p(F6-*co*-PTMSix), respectively. The polymer films were then copiously rinsed with and sonicated in water and TFT for zwitterionic and fluorinated copolymers, respectively, to remove the physically adsorbed polymer and residual traces of unreacted monomers. Films were finally annealed at 130 °C for 1 h.

### 3.3. Water Contact Angle and Surface Chemistry

Static contact angles with water (*θ*_w_) were measured on polymer films covalently grafted to the glass slide and compared with those of the bare glass and the respective homopolymers. Data are collected in Table 1. Copolymers p(F6-*co*-PTMSix) showed values of *θ*_w_ higher than 90° similar to that of the respective homopolymer p(F6) and consistent with the hydrophobic nature of fluorinated (co)polymers [33,34,35]. On the other hand, both MSA and MPC copolymers displayed lower *θ*, even though significantly higher than those of the corresponding homopolymers p(MSA) and p(MPC). This result was unexpected on account of the high content of zwitterionic counits (95 mol%) in p(MPC-*co*-PTMSi5) and p(MSA-*co*-PTMSi5). A moderate hydrophilic nature was previously observed for poly(MPC-*co*-*n*-butyl methacrylate) and poly(MPC-*co*−2-methacryloyloxy-4-azidobenzoate) films with *θ*_w_ values of approximately 60° and 50°, respectively [36]. However, most of the studies in literature showed that zwitterionic copolymers are markedly hydrophilic materials (*θ*_w_ = 15°–30°) [37]. The comparatively high hydrophobicity of the zwitterionic copolymers of this work is possibly due to the surface segregation of low surface energy siloxane groups derived from the condensation between trialkoxysilyl moieties during the covalent grafting to the glass substrate.

**Figure 6:**
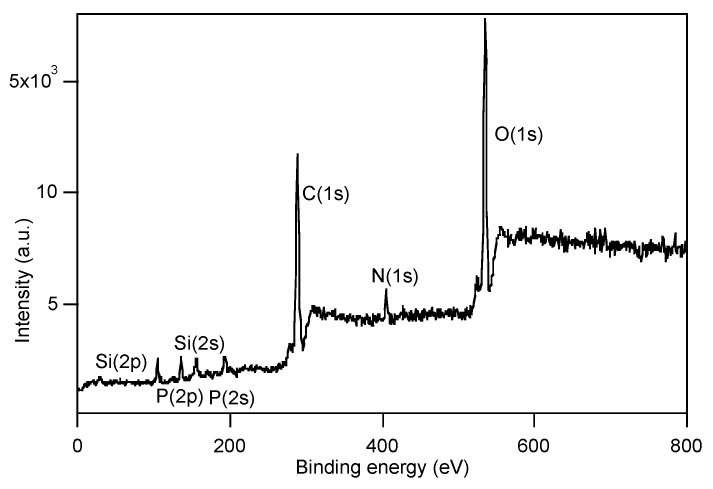
XPS survey spectrum for p(MPC-*co*-PTMSi5) (*ϕ* = 70°).

**Figure 7:**
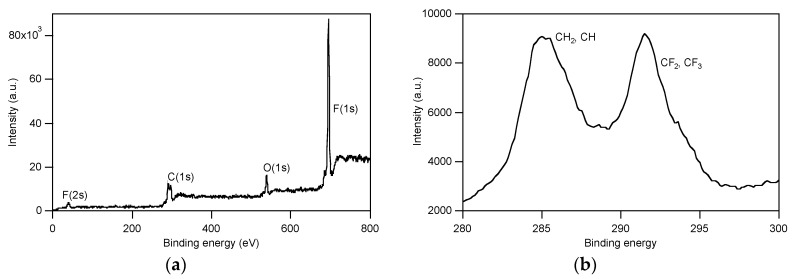
XPS survey (**a**) and high-resolution (**b**) spectra for p(F6-*co*-PTMSi5) (*ϕ* = 70°) after immersion in water for 24 h.

To gain a better comprehension of the film surface composition on account of the findings from biological assays with *C. albicans* (see below, Section 3.4), angle-resolved X-ray photoelectron spectroscopy (AR-XPS) measurements were performed at photoemission angles *ϕ* of 70° and 20° on the films, before and after water immersion for 24 h on the films of copolymers p(MPC-*co*-PTMSi5) (Figure 6) and p(F6-*co*-PTMSix) (Figure 7). The former was also taken as illustration examples of the zwitterionic polymer films for determination of their surface charge by ζ potential measurements at different pH values.

Quantitative XPS data are reported in Table 2. In p(MPC-*co*-PTMSi5) film, the presence of P (P(2p) and P(2s)) and N (N(1s)) moieties at the surface suggests that the glass slides were effectively covalently functionalized with the copolymer (Figure 6). Their atomic percentages were slightly lower than the theoretical ones and did not change significantly by varying the photoemission angle, indicating that the concentration of the phosphorylcholine side chain was essentially constant along the investigated sampling depth. On the other hand, Si (Si(2p) and Si(2s)) was detected with a content that, although relatively low in absolute value, was significantly higher than the theoretical one and decreased by increasing the sampling depth. These results indicate that siloxane groups were concentrated at the outermost surface layers, being the lowest surface energy components in the copolymer system. These findings are consistent with the contact angle analysis and suggest the hypothesis of siloxane bond formation as a result of the condensation of methoxysilyl/silanol groups of PTMSi. Upon immersion in water for up to one week, the surface of p(MPC-*co*-PTMSi5) film still presented Si moieties at the surface and overall the chemical composition after one week of immersion did not differ significantly from that before immersion. This is in contrast to what is normally expected of amphiphilic surfaces in which reconstruction of the outer surface occurs to respond to the changed aqueous outer environment [38]. We speculate that the interchain siloxane groups once formed were stable and did not rearrange upon contact with water.

AR-XPS analysis of p(F6-*co*-PTMSix) films revealed that their surfaces were highly enriched in F (F(1s)) moieties, with a percentage similar for both p(F6-*co*-PTMSi1) and p(F6-*co*-PTMSi5) and higher than the theoretical one, especially at *ϕ* = 70° (Table 2). Moreover, the F percentage decreased with increasing the sampling depth, while C and O percentages followed the opposite trend. Thus, the fluorinated chains were selectively located at the outermost surface layers. For these samples, the amount of Si at the surface was very low, consistent with the theoretical amount in the copolymer. Accordingly, the siloxane groups were segregated in the bulk of the film. Similar remarks are also valid for the films after immersion in water, which presented a slightly increase in Si and a decrease in F concentrations. The F moieties, however, remained the predominant components (Figure 7a). The presence of the resolved peak at ~292 eV in the C(1s) spectrum (Figure 7b) due to the CF_2_ and CF_3_ groups of F6 confirmed that the fluorinated side chains populated the film surface after immersion in water. As a result, no variation in water contact angle after 24 h of immersion was detected (*θ*_w_ = 115° ± 2 and *θ*_w_ = 116° ± 1 for p(F6-*co*-PTMSi5) and p(F6-*co*-PTMSi1), respectively).

The values of electrokinetic ζ potential of coated glass slides with copolymer p(MPC-*co*-PTMSi5) exhibited an ascending trend with decreasing pH and comprised a broad negative plateau (–55 ± 5 mV, pH > 6.5), an isoelectric point (pH = 3.7) and a narrow positive plateau (~20 ± 1 mV, pH < 3). Bare glass slides were negatively charged over the whole pH investigated range (plateau value of –75 ± 2 mV, pH > 4.5) and an isoelectric point at pH = 2.6. Thus, functionalization of the glass surface by MPC was further confirmed, including accumulation of phosphate anion groups at the polymer–water interface. Such values of plateau of ζ potential are consistent with those reported for different phosphorylcholine-modified [39,40] and sulfobetaine-modified [41,42] surfaces. The coated surfaces appeared not to readjust under the adopted experimental conditions, as inferred by the reproducibility on repeated measurements, nor was detachment of polymer film from the glass surface observed in any case. These findings could ensure stability of the tested films during the subsequent biological assays.

### 3.4. Biological Assays with C. albicans

**Figure 8:**
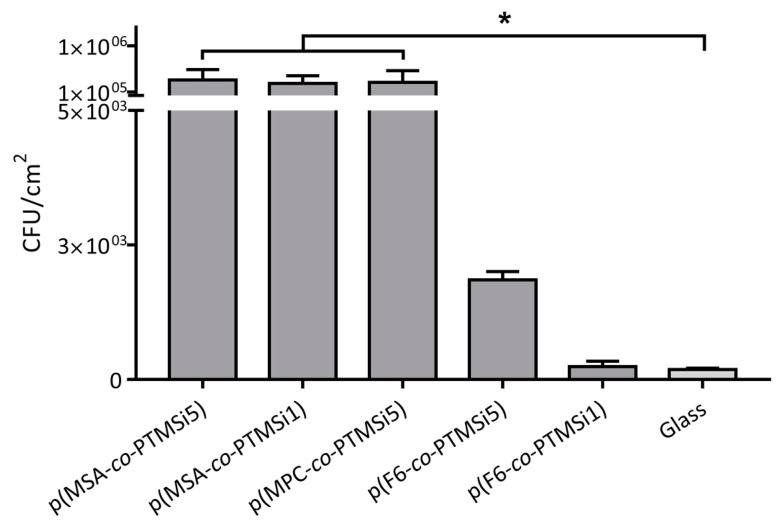
Adhesion of *C. albicans* to zwitterionic and fluorinated films; * *p* ≤ 0.05 (first set of experiments).

**Figure 9:**
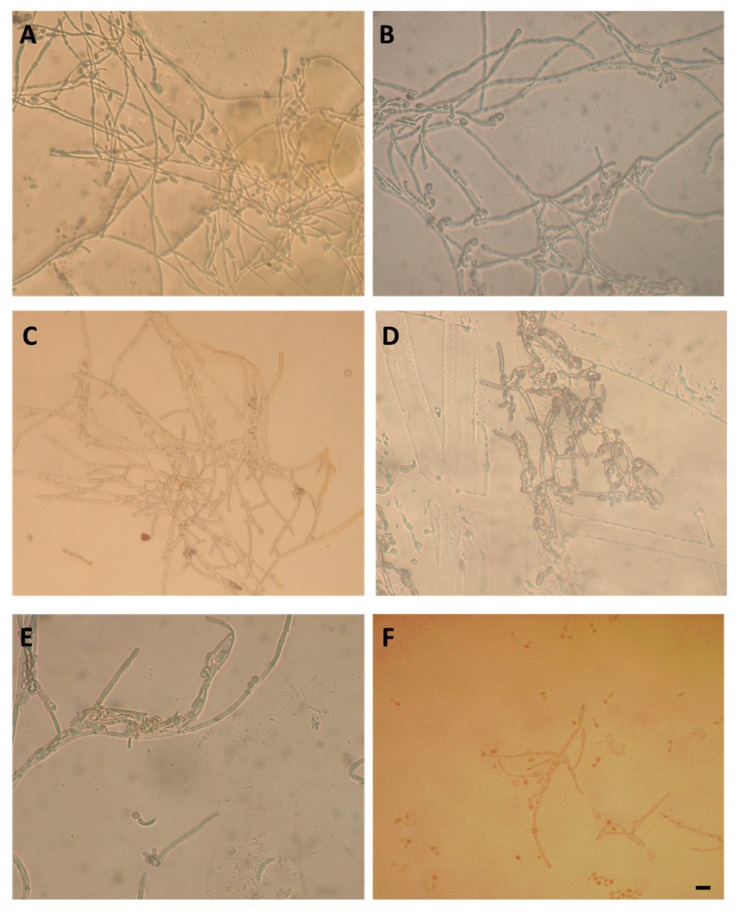
Inverted microscope images (400× magnification) of *C. albicans* SC5314 biofilms grown on different surfaces: (**A**) p(MSA-*co*-PTMSi5), (**B**) p(MSA-*co*-PTMSi1), (**C**) p(MPC-*co*-PTMSi5), (**D**) p(F6-*co*-PTMSi5), (**E**) p(F6-*co*-PTMSi1) and (**F**) glass (scale bar denotes 10 µm for all images).

Results on the ability of *C. albicans* to adhere to, and form biofilm on, the films of zwitterionic and fluorinated copolymers are summarized in Figure 8, which shows the numbers of sessile cells adhered (CFU cm^–2^, surface area 1.8 cm × 1.8 cm) to each slide, following incubation for 24 h to a cell concentration of approximately 2 × 10^7^ cells mL^–1^ for 24 h. Glass surface was also tested as a negative standard, which is known to inhibit the adhesion of *C. albicans*. The results demonstrate a marked reduction in *C. albicans* adhesion on fluorinated surfaces with respect to zwitterionic ones, with the fluorinated p(F6-*co*-PTMSi1) copolymer, showing comparable antifouling performance to that of the negative standard. Inverted microscope analyses of different surfaces revealed the presence of the filamentous forms associated with biofilm formation, with a higher prevalence of hyphal forms on zwitterionic polymers (Figure 9).

**Figure 10:**
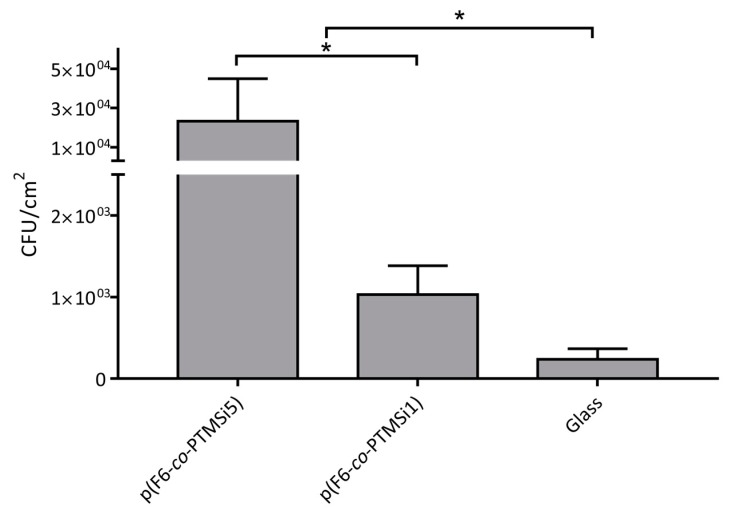
Adhesion of *C. albicans* to fluorinated films; * *p* ≤ 0.05 (second set of experiments).

Since the high variability observed between zwitterionic and fluorinated polymers prevented to assess potential differences in fungal adhesion on fluorinated surfaces with different chemical composition, a second set of experiments of colonization on p(F6-*co*-PTMSix) films was performed to specifically address this point (Figure 10). Oscillations were noticed in the CFU cm^–2^ values, as was expected of the variability of adhesion tests carried out with *C. albicans* at different times. The results confirmed that adhesion of cells on p(F6-*co*-PTMSi1) was significantly lower than on p(F6-*co*-PTMSi5) (1.0 × 10^3^ CFU cm^–2^ vs. 2.3 × 10^4^ CFU cm^–2^), and consistently a higher number of fluorinated units in the copolymer better reduced the adhesion of *Candida*.

## 4. Conclusions

Fluorinated (F6) and zwitterionic (MSA and MPC) copolymers containing low amounts of trimethoxysilyl groups were synthesized, and films therefrom were firmly anchored onto glass slides, for surface functionalization and chemical modification via a “grafting-to” reaction. AR-XPS measurements proved that the grafted polymer films were stable upon contact with water at least up to seven days of immersion, including the water-soluble copolymers p(MPC-*co*-PTMSix). It was also found that the film surface of fluorinated copolymers was largely populated by fluorinated moieties, both before and after immersion in water, consistent with their hydrophobic, low-surface-energy character. However, the film surface of zwitterionic copolymers was enriched in silicon, resulting in a more hydrophobic character than expected of their hydrophilic, high-surface-energy nature.

Biological assays against the most aggressive fungal pathogen *C. albicans* clearly pointed out that fluorinated copolymers were much more able to reduce the adhesion of *C. albicans* than the corresponding zwitterionic copolymers. The amount of F6 counits in the copolymer played a role in decreasing cell attachment, with the copolymer p(F6-*co*-PTMSi1) richer in fluorinated component being the best performer. Therefore, this work helps to overcome some of the discrepancies present in the literature about the role of zwitterionic and fluorinated polymers in preventing the adhesion of *C. albicans* and can provide initial guidelines for the synthesis of polymers with a tailored hydrophilic/hydrophobic balance to combat biofouling. The question of the contrasting “preferences” of biofouling agents to adhere and settle on surfaces is highly relevant in diverse fields of advanced applications. For one example, such fluorinated copolymers might be considered as surface modifiers of biomedical silicones, to be potentially employed in other-than-typical dental materials, to enhance resistance to *C. albicans* colonization.

## Figures and Tables

**Table 1:** Water contact angle for fluorinated and zwitterionic copolymers, the corresponding homopolymers and activated glass.

Film	*θ*_w_ (°)
p(F6-*co*-PTMSi5)	115 ± 2
p(F6-*co*-PTMSi1)	116 ± 3
p(MSA-*co*-PTMSi5)	66 ± 5
p(MPC-*co*-PTMSi5)	69 ± 2
p(F6)	111 ± 1
p(MSA)	10 ± 5
p(MPC)	10 ± 5
Glass	20 ± 3

**Table 2:** AR-XPS atomic surface percentages of p(MPC-*co*-PTMSi5) and p(F6-*co*-PTMSix) before and after immersion in water.

Film		*ϕ* (°)	N (%)	P (%)	C (%)	O (%)	Si (%)	F (%)
p(MPC-*co*-PTMSi5)	before	70	2.8	3.2	59.8	29.5	4.7	-
		20	3.9	3.4	59.5	31.1	2.1	-
		theor.^1^	5.0	5.0	58.1	31.6	0.3	
	after	70 ^2^	2.8	1.8	67.1	23.9	4.4	-
		20 ^2^	4.5	2.6	61.0	29.4	2.5	-
		70 ^3^	1.1	1.2	60.0	26.9	10.7	-
		20 ^3^	3.8	2.6	59.4	30.4	3.9	-
p(F6-*co*-PTMSi5)	before	70	-	-	37.2	6.0	0.2	56.6
		20	-	-	41.9	7.3	0.2	50.6
		theor.^1^			43.0	8.4	0.2	48.4
	after	70 ^3^	-	-	40.1	7.8	1.2	50.9
		20 ^3^	-	-	44.0	8.9	0.7	46.4
p(F6-*co*-PTMSi1)	before	70	-	-	38.2	5.8	0	56.0
		20	-	-	41.9	6.7	0	51.4
		theor.^1^			42.4	7.8	<0.1	49.7
	after	70 ^3^	-	-	39.7	6.6	1.1	52.6
		20 ^3^	-	-	44.2	8.9	0.8	46.1

^1^ Theoretical percentage evaluated on the basis of the stoichiometric composition of the copolymer. ^2^ After immersion for one week. ^3^ After immersion for 24 h.
